# Stabilizing mechanisms in a food web with an introduced omnivore

**DOI:** 10.1002/ece3.2773

**Published:** 2017-06-13

**Authors:** Monica Granados, Sean Duffy, Christopher W. McKindsey, Gregor F. Fussmann

**Affiliations:** ^1^ Department of BiologyMcGill UniversityMontrealQCCanada; ^2^ Fisheries and Oceans CanadaInstitut Maurice‐LamontagneMont‐JoliQCCanada

**Keywords:** adaptive feeding, intraguild predation, invasive species, ontogenetic niche shift, prey size refugia, stage structure

## Abstract

Intraguild predation (IGP) is an omnivorous food web configuration in which the top predator consumes both a competitor (consumer) and a second prey that it shares with the competitor. This omnivorous configuration occurs frequently in food webs, but theory suggests that it is unstable unless stabilizing mechanisms exist that can decrease the strength of the omnivore and consumer interaction. Although these mechanisms have been documented in native food webs, little is known about whether they operate in the context of an introduced species. Here, we study a marine mussel aquaculture system where the introduction of omnivorous mussels should generate an unstable food web that favors the extinction of the consumer, yet it persists. Using field and laboratory approaches, we searched for stabilizing mechanisms that could reduce interaction strengths in the food web. While field zooplankton counts suggested that mussels influence the composition and abundance of copepods, stable isotope results indicated that life‐history omnivory and cannibalism facilitated the availability of prey refugia, and reduced competition and the interaction strength between the mussel omnivore and zooplankton consumers. In laboratory experiments, however, we found no evidence of adaptive feeding which could weaken predator–consumer interactions. Our food web study suggests that the impact of an introduced omnivore may not only depend on its interaction with native species but also on the availability of stabilizing mechanisms that alter the strength of those interactions.

## 1 Introduction

Intraguild predation (IGP) is a specific case of omnivory where predator and prey compete for a common resource. Under IGP, the consumer is subject to strong predatory and competitive interactions and was excluded in early models (Holt & Polis, 1997; Krivan & Diehl, 2005). Yet, dissections of trophic interactions in food webs suggest that this type of omnivory is widespread (Arim & Marquet, 2004; Thompson, Hemberg, Starzomski, & Shurin, 2007) when the interactions in the food web are weak (McCann, Hastings, & Huxel, 1998). Stabilizing mechanisms are thus believed to exist in nature, which alter the strength of interactions between constituents of the food web (Polis, Myers, & Holt, 1989). Kratina, LeCraw, Ingram, and Anholt (2012) partition these stabilizing mechanisms into five main categories: habitat complexity, antipredator phenotypes of prey, adaptive feeding behavior of omnivores, life‐history omnivory, and cannibalism. In each, the stabilizing mechanism reduces the probability of consumption of the prey thus altering the strength of the interaction. While stabilizing mechanisms have been documented in native food webs (Finke & Denno, 2002; Janssen, Sabelis, Magalhães, Montserrat, & van der Hammen, 2007; Rickers, Langel, & Scheu, 2006; Rudolf & Armstrong, 2008), very little is known about their existence in food webs with introduced omnivores. There is reason to believe, however, that the lack of evolutionary history between the introduced omnivore and the recipient community should favor the absence of stabilizing mechanisms. Tantamount to the naïve prey hypothesis, where the lack of evolutionary history precludes adaptation of naïve prey to introduced predators resulting in ineffective antipredator behavior, allopatry among species in a food web may prohibit the presence of stabilizing mechanisms (Sih et al., 2010). Most stabilizing mechanisms, including four of the five major categories in Kratina et al. (2012), arise through the selection of traits that favor a reduction in interaction strength and an increase in food web stability. For example, the evolution of scale armor has been shown to decrease the consumption of threespine stickleback by its sympatric omnivorous predator, contrary to allopatric stickleback which were consumed a higher rates (Ingram et al., 2012). Yet, there exists the possibility that stabilizing mechanisms could be present in a recipient community in the absence of selection if traits conducive to stabilizing mechanisms are pre‐existing in the resident species and the introduced omnivore.

**Figure 1:**
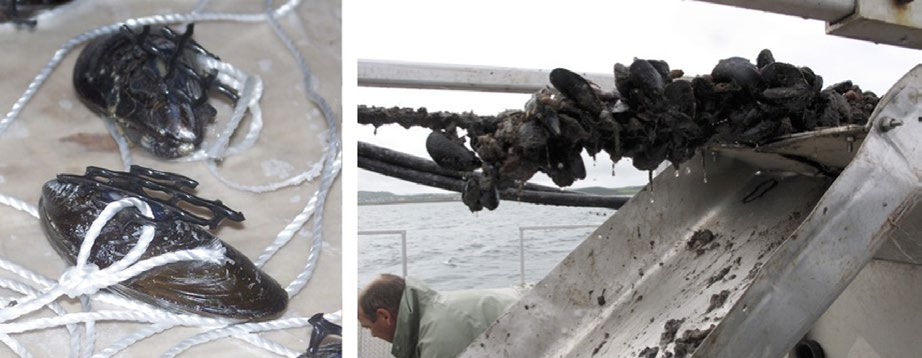
Photographs of *Mytilus edulis* mussels being prepared for feeding experiment (left) and harvested off a line (right). Photographs by Sean Duffy

Because interaction strength governs whether omnivory has a positive, stabilizing impact or a deleterious extinction effect in food webs, the presence of stabilizing mechanisms that reduce interaction strength may facilitate the persistence of the consumer. If an introduced omnivore forms strong interactions with the native consumer and its shared resource, its introduction can lead to the extinction or reduction in consumers and resources (Hall, 2011a). A notable example is the introduction of the omnivorous rusty crayfish, *Orconectes rusticus—*introductions of this species have directly led to the decline of both macroinvertebrate consumers and shared common resources (Lodge, Kershner, Aloi, & Covich, 1994; McCarthy, Hein, Olden, & Jake Vander Zanden, 2006). Recently, strong functional responses, which measure the intensity of predation, have also been positively associated with greater impacts among introduced omnivores in recipient communities (Dick et al., 2014). Here, we examine the introduction (~30 years ago) of the omnivorous blue mussel, *Mytilus edulis* (Figure 1), to the Havre‐aux‐Maisons Lagoon (HAM) for the purposes of aquaculture (Richard, Archambault, Thouzeau, & Desrosiers, 2006). The blue mussel is generally regarded as a microphagous filter feeder. However, it also has the capacity to consume zooplankton, where pre‐adult life stages are most vulnerable to predation (Jonsson, Nielsen, Hrubenja, Maar, & Petersen, 2009; Lehane & Davenport, 2006). Significant ingestion of zooplankton by mussels has been recorded both experimentally with *Artemia* nauplii as proxies (Davenport, Smith, & Packer, 2000) and in natural systems (Nielsen & Maar, 2007). Because naturally occurring mussels are benthic organisms, confined to consuming resources present in the water above substrates, the potential for competition and predation between mussels and zooplankton is limited (Maar, Nielsen, & Petersen, 2008). In contrast, off‐bottom or suspended mussel aquaculture places mussels in the water column using a series of lines as substrate that span the upper reaches of the pelagic zone (Lehane & Davenport, 2002; Maar, Nielsen, Bolding, Burchard, & Visser, 2007). Under aquaculture conditions, where dense mussel populations are in contact with zooplankton, the potential for omnivory becomes appreciable. Suspended in lines, the mussel operates as the omnivore which consumes and competes with zooplankton and micro/nanoplankton (i.e., phytoplankton, heterotrophic protists; seston) are the common resource. Hereafter, we refer to mussels as the omnivore the zooplankton (copepods) as the consumer and the seston as the common resource. In this study, we use this mussel aquaculture food web to 1. determine whether consumers persist at sites where the omnivorous mussel has been introduced and 2. search for stabilizing mechanisms that may be reducing all the interaction strengths in the food web (omnivore–consumer, consumer–resource, omnivore–resource). Given that mussels can selectively filter‐feed by increasing feeding currents and valve gapes (Gosling, 2003; Riisard, 1991) and zooplankton growth proceeds in distinct ontogenetic stages (Johnson & Allen, 2005), we tested for the presence of adaptive predator feeding behavior, life‐history omnivory, and cannibalism stabilizing mechanisms.

Interaction strengths in the food web can be mediated through age/stage structure and ontogeny where omnivorous interactions are subject to change as both prey and predator increase in size or age during the course of their development (Browne & Rasmussen, 2009; Olson, Mittelbach, & Osenberg, 1995; Rudolf & Armstrong, 2008). Stage structure in food webs can beget life‐history omnivory and cannibalism, both which can lead to a reduction in interaction strength (Kratina et al., 2012). In life‐history omnivory, adult omnivores prey on consumers, while juvenile omnivores compete with consumers for a common resource, thereby reducing the interaction strength between some omnivore and consumer life stages (Hin, Schellekens, Persson, & de Roos, 2011). Cannibalism, although usually discussed as a behavior of the omnivore, can be expressed in the consumer and results in adult consumers preying on juvenile consumers. As a density‐dependent process, cannibalism reduces the per capita interaction strength between consumer and resource and dampens consumer‐resource population cycles (Kratina et al., 2012; McCann, 2012).

Adaptive feeding is defined as a diet modification by the omnivore based on the profitability and abundance of its two resources, either by switching prey or by adjusting the proportion of each in a mixed diet (McCann, Rasmussen, & Umbanhowar, 2005). The behavior is adaptive because less energy is required to prey on the resource at higher density. Adaptive feeding also releases the prey from predation when either consumer or resource density is low, allowing it to recover and stabilizing the food web (Gismervik & Andersen, 1997; Krivan & Diehl, 2005; McCann, 2012).

We first sampled the HAM to assess consumer abundance and composition between farm sites and areas where farming has not occurred (reference sites). In the HAM, we also analyzed the stable isotope signature of mussel and plankton samples to determine whether the strength of predation changed with mussel size and whether adult stages of the consumer experienced a size refugium that affords them reduced predation. Because nitrogen turnover rate in *Mytilus edulis* is over 40 days and zooplankton and seston data are often used as base lines, stable isotope data can inform trophic relationships in the HAM (Dubois, Jean‐Louis, Bertrand, & Lefebvre, 2007; Hawkins, 1985; Post, 2002). Finally, we performed a laboratory experiment to investigate how mussels consume a common resource versus a consumer—that also feeds on the common resource—and whether they exhibit prey switching. We tested the hypothesis that omnivory in mussel aquaculture food webs depends on the relative abundance of consumer and common resource by offering varying proportions of these two prey types and by quantifying their relative uptake using selectivity indices.

## 2 Materials and Methods

### 2.1 Copepod community stage structure

Our field site was located in the HAM, Îles de la Madeleine, Quebec. The surface area of HAM is 30 km^2^, with a mean depth of 3 m and about 5–6 m in the aquaculture sites (Richard et al., 2006). The tides are small (ca. 0.6 m), and frequent strong winds drive water mixing and renewal (Minagawa & Wada, 1984). The lagoon is currently used for long‐line mussel aquaculture. We selected two sites within the mussel aquaculture farm and two reference sites outside the farm to characterize the consumer—here zooplankton—community and assess the impact of mussel grazing. Individuals in the subclass copepoda dominate the HAM zooplankton community, and thus, we limited the characterization of the community to copepods (Cherif et al., 2016). We collected copepods using a 73‐μm plankton net, with a diameter of 50cm, towed to a depth of one meter in August 2009. Because distance to the omnivore could influence copepod densities, each farm site was sampled three times between lines and next to the line and six tows were performed in reference sites (Nielsen & Maar, 2007). We thus obtained a total of six tows for each farm and reference site. We identified copepods to genus and classified them to one of three stage classes: adult, copepodite, or nauplii under a dissecting microscope, data which formed the copepod community stage structure data. Given there was no difference between next to the line and in‐between‐line sampling, we used each tow as a replicate for the multivariate analyses while removing tows with many zeroes for a total of nine farm and seven reference tows (*p* = .883, ANOVA). Count data were used to produce a two‐dimensional ordination plot using nonmetric multidimensional scaling (NMDS) and a Bray‐Curtis dissimilarity with site as a grouping factor. Subsequently we used the Bray‐Curtis dissimilarity to perform a SIMPER analysis using the vegan package in R to determine the contribution of each copepod stage structure to the Bray‐Curtis dissimilarity (Oksanen et al., 2005). We also conducted an analysis of similarities (ANOSIM) with site as the grouping factor to quantify the between and within group similarities. Count data were converted to density of individuals L^−1^ and pooled to site level. We performed a two‐way ANOVA on the density data with site (farm or reference) and stage class as fixed factors to assess differences in copepod density between sites and across age classes. Differences between site and stage class were analyzed using Tukey's HSD post hoc test. All statistical analyses were performed with R (R Core Team 2016).

### 2.2 Trophic position field study

Generally, there are three age classes of mussels (0+, 1+, and 2+ years old) growing in different demarcated areas of the farm. Each of these sites contains one of the three age classes. We randomly sampled the mussels in site 1+ and site 2+ which contain larger size range of mussels and are located adjacent to each other in the farm. *Mytilus edulis* were collected off mussel socks on 23 August and 2 September 2010. Throughout this period, multiple plankton net tows (75 μm) and water samples (<60 μm, using a Niskin bottle) were taken at 3 m depth from random positions within sites of the farm dedicated to the different age groups of mussels. Tow contents were immediately frozen for subsequent isotope analysis.

Each mussel collected was measured in mm (shell length) after which the mantle tissue was dissected. Seston <60 μm was collected on GF/F filters (precombusted at 450°C for 12 hr) by filtering one liter of each water sample. Copepods from the plankton net tows were separated into adult and naupliar stages under a dissecting microscope. Nauplii were concentrated onto precombusted GF/F filters to accumulate sufficient organic material. All samples were dried in a lyophilizator for at least 24 hr.

The stable isotope signatures (δ^13^C and δ^15^N) for *Mytilus edulis* mantle tissue, adult copepods, nauplii, and seston were measured by the University of New Hampshire Stable Isotope Laboratory using a Delta Plus XP Mass Spectrometer interfaced to a Costech ECS4010 Elemental Analyzer. The δ^13^C and δ^15^N values are expressed as deviations from a standard in parts per thousand (‰) and calculated as

(1) $$\delta^{13}\text{C or}\;\delta^{15}\;N=\left[\left(R_{\mathrm{sample}}-R_{\mathrm{standard}}\right)/R_{\mathrm{standard}}\right]\times 10^{3}$$

where *R* is the ratio of ^13^C/^12^C or ^15^N/^14^N. Trophic position is directly related to δ^15^N (Minagawa & Wada, 1984; van der Zanden & Rasmussen, 2001), whereas the source of the food is generally determined by similarities in δ^13^C signatures. Lipids are depleted in ^13^C, and variable lipid storage between species can alter the interpretation of δ^13^C values (McConnaughey & McRoy, 1979). Post et al. (2007) showed that lipid content is strongly related to C:N ratios for aquatic organisms. The δ^13^C signatures of all our samples were corrected for lipids using the following equation from Post et al. (2007):

(2) $$\delta^{13}C_{\mathrm{normalized}}=\delta^{13}C_{\mathrm{untreated}}-3.32+0.99\times \text{C:N}$$

where C:N is the mass ratio of carbon and nitrogen in the sample. Although the authors suggest a different equation to correct for lipids in photosynthetic organisms, we applied equation (2) to the δ^13^C values of the seston as we considered the samples to be mainly heterotrophic protists (Trottet, Roy, Tamigneaux, & Lovejoy, 2006). δ^15^N values are not affected by lipid content and do not require transformation. For site 1+, we processed a total of 57 mussel, 15 adult copepod, 18 nauplii copepod, and 14 seston samples. For site 2+, we processed a total of 64 mussel, 11 adult copepod, 19 nauplii copepod, and 15 seston samples.

δ^15^N data were tested for homogeneity of variance (Levene's test) and normality (Shapiro–Wilk test) to satisfy the assumptions of parametric statistical analyses. We analyzed the difference between the δ^15^N signature of mussels and potential prey items by calculating the mean δ^15^N for each prey tissue type and the 95% confidence intervals (CI) around each mean for each site. To determine whether larger mussels had higher δ^15^N than copepod nauplii, we split mussels into two categories—greater or less than the upper bound 95% CI of the mean δ^15^N for copepod nauplii. We selected the higher δ^15^N bound between the two sites to have a more conservative estimate of the upper bound. This new binary variable was then used to generate a logistic regression using a generalized linear mixed model fit by maximum likelihood using a binomial distribution with site as a random factor. The logistic regression describes the relationship between mussel length and the probability that mussel δ^15^N is greater than copepod nauplii δ^15^N. To quantify this relationship, we calculated the exponent of the slope coefficient. As the trophic position of blue mussels was our primary interest, δ^13^C data were not included in the statistical analysis and are just presented to support the δ^15^N data (Table S3 in Appendix S1). All statistical analyses were performed with R (R Core Team 2016).

### 2.3 Adaptive feeding laboratory experiment

The experiment was performed at the Maurice Lamontagne Institute in Mont‐Joli, Quebec, Canada. *Mytilus edulis* (shell length range 53 ± 3 mm) were obtained from an aquaculture farm in baie des Chaleurs (Carleton, Quebec). Mussels were maintained in controlled conditions prior to experimental trials; each mussel was glued to a line and suspended in 200 liter flow through basins supplied with unfiltered raw St. Lawrence estuary seawater (6–9°C) taken from off‐shore of the research institute (Figure 1). A nonaxenic strain of the flagellated alga, *Isochrysis galbana* (Prymnesiophyceae, supplier: NutrOcéan), was used as the common resource. Algae were grown in 30 liter batch cultures at 21**°**C using Guillard's f/2 medium (Guillard, 1975). The cultures were drained every 3 days and replenished with fresh medium to keep the algae in an exponential phase of growth. The average cell diameter of the algae was 6.1 μm, and the dry weight was 5 × 10^−5^ μg/cell (estimated from Fidalgo, Cid, Torres, Sukenik, & Herrero, 1998). The diameter of the algae is greater than the minimum size accepted by the inhalant siphon of the mussel (Gosling, 2003) and at <60 μg well within the size range of the seston in the stage structure composition study. Nauplius larvae of *Artemia franciscana* (Anostraca, Crustacea) were used as the consumer in the experiment (<24 hrs old). *Artemia* nauplii were used as proxies for copepods *in situ* due to their comparable sizes and behavior in the inhalant siphon of mussels (Davenport et al., 2000). Nauplii were hatched daily by suspending eggs in aerated filtered seawater 22 hours before the start of experimental trials. Un‐hatched eggs were siphoned off from the cultures. The average length of *Artemia* nauplii was 454 μm, and the dry weight was 1.0 μg/individual, estimated from Abreu‐Grobois, Briseno‐Duenas, Herrera, and Malagon (1991).

Twelve‐liter plastic buckets filled with 8 L of 0.2 μm UV‐treated seawater (6**°**C, salinity = 26 PSU) each received one mussel. Each mussel was suspended in the center of an experimental bucket by hanging it from a dowel resting across the rim. Mussels were provided one of seven diet treatments varying in biomass proportion of *algae:Artemia* (100:0, 90:10, 75:25, 50:50, 25:75, 10:90, 0:100). The total biomass of each diet treatment was the same (4,000 μg dry weight). To prepare diet mixtures, we estimated the densities of algae and *Artemia* cultures by counting the individuals from a sub‐sample using a Neubauer hemocytometer. From this, we calculated the volume needed from each culture to make up the diet treatments. Control buckets without mussels received the diet mixture as well. Diet treatments were replicated six times for buckets with and without mussels (84 buckets total). Although we aimed for the diet proportions listed above during preparation, the actual diet mixtures measured at the start of the experiment were used for the analysis. The experimental trials were run for one hour in an incubated room at 9**°**C without light to avoid stimulating algal growth and altering *Artemia* swimming behavior. Gentle aeration kept the water in each bucket homogenized. The mussels were acclimated to their experimental conditions for a 24‐hr period prior to the initiation of each trial. During this acclimation period, mussels were fed their prescribed experimental dietary treatment.

At the start and end of experimental runs, we took 10 mL water samples from all treatment and control buckets and measured raw fluorescence using a Turner Designs Trilogy Fluorometer. Raw fluorescence values were converted using a standard curve to estimate the density of algae. *Artemia* were collected at the end of the experiment by straining the contents of each bucket using a 64 μm filter and fixing them in 70% ethanol. These samples were counted under a dissecting microscope to determine *Artemia* density.

Clearance rate on algae and *Artemia* was used to measure the grazing activity by mussels and is defined as the volume of water cleared of a given prey type per unit time per mussel (volume time^−1^ mussel^−1^). Specific clearance rate (*F*_i_) for the ith prey type was calculated according to Coughlan (1969) as

(3) $$F_{i}=\left(V/\Delta t\right)\;\left(\ln\;\left[C_{0i}/C_{\mathrm{ti}}\right]\right)$$

where *V* is the volume of water in each bucket and *Δt* is the elapsed time. When calculating clearance rates for algae (*F*_Iso_), *C*_0i_ and *C*_ti_ are the algal concentrations at time 0 and time *t* in buckets with mussels. To account for algal growth and sedimentation, we calculated the mean clearance rate on algae in control buckets with no mussels for each diet mixture and subtracted the corresponding mean from each *F*_Iso_ value of the same diet. We intentionally used nauplii <24 hr old which age they have not developed feeding morphology and instead use yolk reserves. Therefore, consumption by zooplankton is negligible given newly hatched *Artemia* do not feed (Heath, 1924). For clearance rates on *Artemia* (*F*_Art_), *C*_0i_ and *C*_ti_ are the nauplii concentrations in control and treatment buckets, respectively. We assumed no growth in the *Artemia* population given the short duration of the experiment and the molting rate of *Artemia* (Chesson, 1978).

The selectivity coefficient (*S*_i_) for the ith prey type was calculated as

(4) $$S_{i}=F_{i}/\sum F_{i}$$

where a value of *S*_i_ above or below 0.5 indicates feeding preference or avoidance, respectively (Chesson, 1978; Vanderploeg & Scavia, 1979). Changes in selectivity across diets were analyzed using linear regression. If the regression was not significant, the selectivity data from all mixed diets were pooled and the t‐statistic was used to test for significant deviations from random feeding (*S*_i_ = 0.5). Selectivity data met the assumptions of normality (Shapiro‐Wilk test) and homogeneity of variance (Breusch–Pagan test). All statistical analyses were performed with R (R Core Team 2016).

## 3 Results

### 3.1 Copepod community stage structure

**Figure 2:**
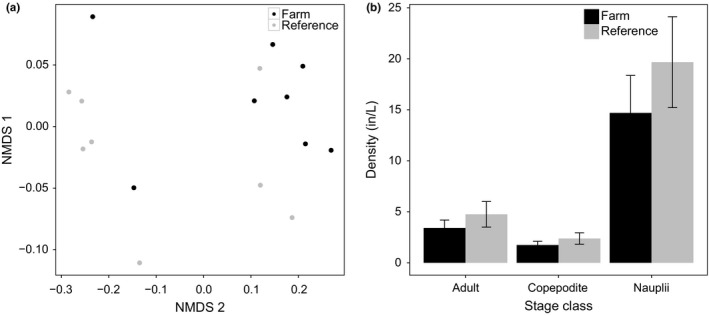
(a) NMDS plot of farm and reference sites based on of zooplankton species and life‐stage composition. ANOSIM result indicates the composition between the two sites is significantly different (*p* = .045). B. Bar plot of zooplankton density in farm and reference sites for each zooplankton life stage. Error bars denote standard error. Reference sites had significantly higher densities (ANOVA, *p* = .031)

The ordination qualitatively demonstrated that the copepod communities between farm and reference sites were distinct (Figure 2a). In an ordination, where distance on the plot is inversely related to similarity, farm sites group together and reference sites are closer to each other than to farm sites. The ANOSIM indicated that copepod community stage structure was significantly different between farm and reference sites (*p* = .045, *R* = .204). The SIMPER analyses suggest that these differences are driven by Acartia nauplii given their average contribution to the average dissimilarity is greatest. (SIMPER, 19% average contribution) followed by *Tmora* nauplii (SIMPER, 6.7% average contribution, Table S1 in Appendix S1). The ANOVA results on the density data indicated pooled copepod density was greater in reference sites (Figure 2b, ANOVA, *p* = .031, Table S2 in Appendix S1).

### 3.2 Trophic position field study

**Figure 3:**
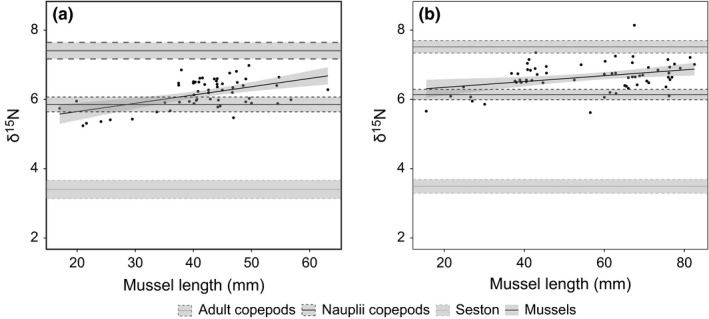
Plot of δ^*15*^N against mussel size fitted with a linear model (δ^*15*^N ~ mussel length) for A. Site 1+ and B. Site 2+. Horizontal lines are the mean δ^*15*^N for adult copepods, copepod nauplii and seston in dark gray, black, and gray, respectively with 95% confidence intervals plotted around the means in dashed lines

**Figure 4:**
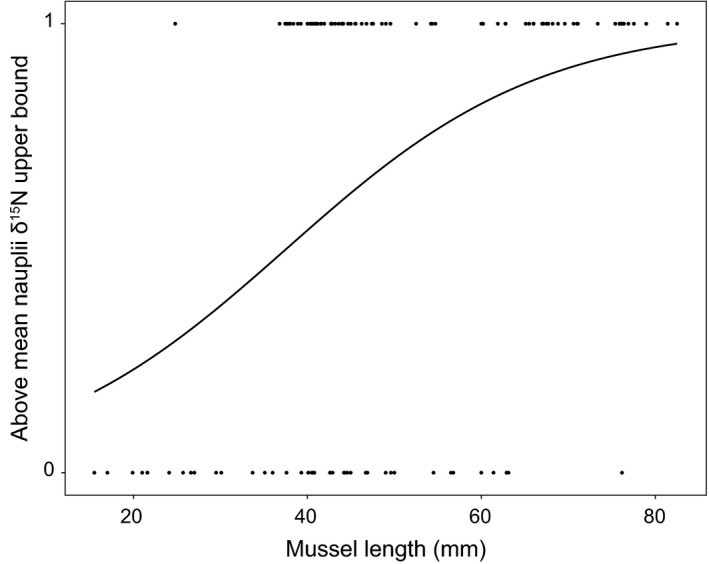
Logistic regression of mussel length and a binary variable created by assigning mussels into categories based on whether their δ^*15*^ *N* value was greater (1) or less than (0) the upper bound of the 95% confidence interval around the mean δ^*1*5^N of copepod nauplii. A one‐unit increase in mussel length increases the probability the mussel δ^*15*^N will be greater than nauplii δ^*15*^N by 1.06 (GLMM)

Clear trophic structure was demonstrated by the δ^15^N data with seston at the base of the food web, followed by copepod nauplii and adult copepods occupying the highest trophic position at both site 1+ and 2+ (Figure 3). Mussels demonstrated a linear increase in δ^15^N with length—with smaller mussels feeding closer to the base of the food web and larger mussels farther up, but feeding lower than adult copepods. Results from the linear regression looking at intraspecific changes in trophic position revealed a significant positive relationship between δ^15^N and mussel shell length for both sites 1+ and 2+ (site 1+ *R* ^2^ = .254, *p* < .001, site 2+ *R* ^2^ = .104, *p* < .001), indicating that larger mussels occupied a higher trophic position (Figure 3). In both site 1+ and 2+, the seston mean and CI did not overlap with the mean and CI of any of the other tissue types. In site 1+, no mussel had a greater δ^15^N than the lower bound of the CI around the mean adult copepod δ^15^N and in site 2+ only two mussels had a greater δ^15^N than the adult copepod lower bound. The logistic regression of mussel length and the binary variable created by categorizing mussel δ^15^N by the nauplii CI upper bound indicates a positive relationship between mussel length and likelihood of the δ^15^N value being greater than the copepod nauplii δ^15^N (Figure 4). The exponent of the logistic regression of 1.06 indicates that the probability that the δ^15^N of mussels is greater than the δ^15^N of nauplii increases by 1.06 for every increase by one millimeter in mussel length.

The mean (±SD) δ^13^C signatures from the original dataset widely ranged from −24.61 ± 0.47‰ (seston < 60 μm) to −20.60 ± 0.51‰. After mathematical lipid correction, δ^*13*^ *C* values increased and became more aligned, with the farthest outlying seston values being shifted the most (Table S3 in Appendix S1). This lends credence to the assumption that all the organisms analyzed were part of one food chain supported by the same carbon pool. The sample variance in δ^*13*^ *C* values was amplified because of lipid correction as well.

### 3.3 Adaptive feeding laboratory experiment

Our target‐feeding ration in each bucket was 4,000 μg of total biomass (dry weight). However, based on the initial densities of *Artemia* and algae, the estimated mean (±SE) total biomass per bucket was 4,588 ± 110 μg. On average, 60–70% of the prey populations remained at the end of each experimental run. We found no pseudofeces in the containers at the end of each experimental run, indicating that mussels successfully ingested all the *Artemia* nauplii that were removed from the buckets.

**Figure 5:**
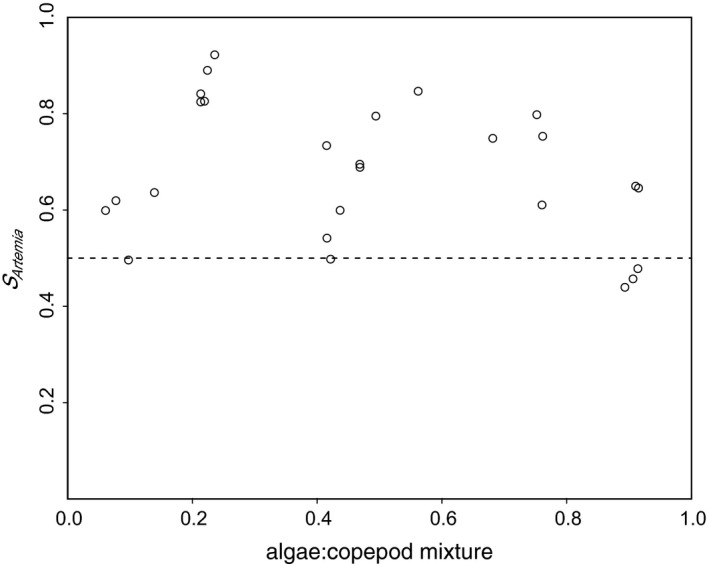
Selectivity of mussels (*Mytilus edulis*) for nauplii (*Artemia franciscana*) (*S*_Artemia_) in feeding trials. Diets with different fractions of algae (*Isochrysis galbana*) vs. nauplii were offered (fraction values are based on proportion of biomass). Total biomass of the algae and nauplii was constant across all mixtures. The dashed line denotes no feeding preference by mussel for either prey. S_Isochrysis_ values are not shown as they mirror *S*_Artemia_ values (*S*_Isochrysis_ = 1 − *S*_Artemia_), and the regression result is the same for both

The mean (±SE) specific clearance rates on algae and *Artemia* from unmixed diet treatments (i.e., 100% algae or 100% *Artemia*) were 2.24 ± 0.41 L h^−1^ mussel^−1^ and 4.52 ± 0.59 L h^−1^ mussel^−1^, respectively. Selective feeding appeared to decrease with increasing proportions of algae, although this trend was not significant (*R* ^2^ = .094, *p* = .127) (Figure 5). When pooling the selectivity data from all mixed diets, mussels exhibited significant deviations from random feeding (*t* = 6.43, *p* < .001) with *Artemia* taken up in greater proportions than what was offered (mean ± SE of S_Artemia_ = 0.69 ± 0.03) (Figure 5).

## 4 Discussion

The present study combined field sampling and laboratory experimentation to characterize the consumer prey of omnivorous mussels in the HAM, assess ontogenetic niche shifts in this food web and the presence or absence of prey switching. In our experiment, we established that mussels have a significant impact on copepod communities *in situ*, that *Artemia* can even be positively selected over the common resource and that the preference for *Artemia* does not change with the relative proportion of the two prey types. While we did not detect adaptive feeding in our experimental system, we identified potential stabilizing mechanisms in the *in situ* sampling. Here, stable isotope signatures revealed life‐history omnivory and associated prey size refugia, cannibalism between adult copepods and nauplii along with ontogenetic niche shifts of the consumer. These coexistence mechanisms essentially weakened the interaction strength in the food web. We propose these coexistence mechanisms potentially allow the zooplankton to sustain itself as postulated by theory (Kratina et al., 2012).

### 4.1 Adaptive feeding by mussels

Our experiment showed that the mussels were selectively ingesting *Artemia* and “avoiding” algae; that is, for all experimental algae:*Artemia* ratios *Artemia* was taken up in higher proportions than present in the prey mix (Figure 5). More importantly, mussels showed no evidence for adaptive feeding, that is, mussels consistently preferred *Artemia* nauplii over algae, independent of the ratio of the two prey objects (Figure 5). There was a tendency toward increased selectivity for *Artemia* with higher *Artemia* proportions in the prey mix, but this trend was not statistically significant. Consequently, we did not detect a behavioral coexistence mechanism in mussels. Both Gismervik & Andersen (1997) and Krivan & Diehl (2005) found that adaptive feeding improved the conditions for the consumer to persist; however, this behavior appears to be absent in our experimental omnivorous food web. We extrapolate these results to the *in situ* food web where these manipulations would prove exceedingly difficult to perform. We follow the example of Davenport et al. (2000) who also used *Artemia* as a proxy for copepods to study mussel predation. In that study, mussels were shown to have similar pumping rates when ingesting *Artemia* versus copepods from the environment. Further, *Artemia* and copepod escape success was analogous in their experiments. We submit, therefore, that *Artemia* are good proxies for HAM copepods due to similar escape behavior, mussel feeding response, and ease of culturing. While adaptive feeding and superior competitive ability is absent in this food web, ontogenetic niche shifts and coincident prey size refugia were successfully detected.

### 4.2 Copepod community stage structure

Field data indicated the presence of mussels have a very real and significant impact on copepods in the HAM. The reduction in copepod density and difference in stage structure composition in the farm sites suggests that mussels are indeed participating in omnivory. Although it is likely that the copepod community is benefiting from migration events from sites where mussels are absent, the presence of all three stage classes in farm sites suggests the persistence of zooplankton cannot be explained simply by source‐sink dynamics (Figure 2b). The nauplii that are produced from adults that emigrate from reference to farm sites are reaching the copepodite stage, suggesting the presence of other stabilizing mechanisms that are weakening predation on nauplii. Additionally, the difference in stage structure composition between farm and reference sites suggests that migration events are not replacing the individuals lost through predation across all stage classes.

### 4.3 Trophic relationships

#### 4.3.1 Life‐history omnivory

In the mussel aquaculture food web, the consumer benefits from not only a reduction in predatory interactions through life‐history omnivory but a reduction in competition between the omnivore and consumer. Stage‐structure limited predatory interactions to the largest mussels with the earliest larval stages of the copepods. The elevated δ^15^N signatures in larger mussels suggest they are consuming considerable amounts of copepod nauplii (Figure 3). This result is consistent with the stage‐structure composition data which indicated the differences between farm and reference sites were most marked in nauplii. Additionally, the significant positive relationship between δ^15^N and mussel shell length (Figure 3) suggests a gradual shift toward stronger omnivory as farmed mussels increase in size. Here, life‐history omnivory creates a size refuge for adult copepods. This size refugia allows potential prey to outgrow predation (Hin et al., 2011), and in food webs with omnivory, prey refugia can decrease the interaction strength between predator and prey as well as the niche overlap with competitors (Woodward & Hildrew, 2002). The isotope data indicate that adult copepods were rarely (or never) ingested by mussels despite being very well represented in aquaculture farms (Cherif et al., 2016). The calanoid *Acartia* sp. is the dominant large zooplankton species of HAM (Cherif et al., 2016) and is a copepod with a strong escape response, even in the naupliar form (Green, Visser, Titelman, & Kiørboe, 2003; Titelman, 2001). *Acartia* adults experience significantly lower predation rates by mussels compared to other copepods (Jonsson et al., 2009; Lehane & Davenport, 2006).

Stage structure in copepods also reduced competition between adult copepods and mussels. The smallest size class of mussels and copepod nauplii has similar δ^15^N signatures and likely compete for seston, while the larger mussels were at a significantly higher trophic level (Figure 3). Limiting competitive interactions for seston to juvenile mussels and copepod nauplii effectively reduced the interaction strength between the omnivore and the resource as less individuals are competing for the same resource.

#### 4.3.2 Cannibalism

Adult copepods sampled in this study tend to feed on dinoflagellates, other algae, ciliates, and copepod nauplii (Lonsdale, Heinle, & Siegfried, 1979). Adult copepods occupied a higher trophic position than the largest mussels (Figure 3). As copepods cannot prey on mussels, the result suggests that organisms from higher trophic levels, such as nauplii, make up a greater proportion of the adult copepods’ diet than of the large mussels. Cannibalism between copepods stages increases the mortality of the consumer which acts to decrease the coupling strength (flux of material between consumer‐resource) relative to the loss terms (mortality). This stabilizes the consumer–resource interaction by limiting the growth of the consumer population and consequently how quickly the consumer can depress the resource density. Effectively the addition of adult copepods as predators mutes oscillations between the consumer and resource (McCann, 2012).

The greater reliance on copepod nauplii also suggests that adult copepods have less niche overlap with larger mussels, and therefore, the interaction strength between consumer and resource is also reduced. Ontogenetic niche shifts, as demonstrated above, have been shown to promote the maintenance of omnivory both empirically (Rudolf & Armstrong, 2008) and theoretically (Hin et al., 2011) by increasing the productivity range where the food web can persist. A previous study found the density of the common resource in Îles de la Madeleine is high (Trottet et al., 2006). High productivity or high density of the common resource should exclude the consumer because the potential competitive superiority of consumer is rendered moot when the resource is not limiting (Mylius, Klumpers, de Roos, & Persson, 2001). However, the presence of stage structure reduces interaction strengths which allow consumers to persist even under high resource density in the HAM.

We conclude that stage structure allows for life‐history omnivory and cannibalism between consumers which reduces the interaction strength between all the components of the food web. This weakening of interactions is likely contributing to the persistence of the consumer in the HAM as neither competition nor predation by the omnivore is high.

### 4.4 Implications for introduced species

Initially, the introduction of an omnivore with its simultaneous predation and competition would appear to be devastating to recipient communities, particularly consumers. While there are many documented introductions with deleterious effects, as in native food webs, stabilizing mechanisms could reduce interaction strengths in food webs with introduced omnivores (Dick & Platvoet, 2000; Hall, 2011a,b; Lodge et al., 1994). Further, our study suggests that these coexistence mechanisms need not necessarily evolve if the traits that form these stabilizing mechanisms are pre‐existing and that these stabilizing mechanisms may reduce the impact of the introduced omnivores facilitating for the persistence food webs, generally.

Species introductions have the power to radically change recipient ecosystems and can force population declines, species extirpations, and extinctions (Bellard, Cassey, & Blackburn, 2016; Blackburn, Cassey, Duncan, Evans, & Gaston, 2004; Clavero & García‐Berthou, 2016). Curtailing these impacts requires a predictive understanding of impact (Ricciardi, Hoopes, Marchetti, & Lockwood, 2013). Here, we show that an understanding of factors that can alter the strength of the novel interactions formed by the introduced species is necessary to assess the consequences of the introduction of an omnivore and the impact of aquaculture.

## Conflict of Interest

None declared.
