# Supplementary material for: Stabilizing mechanisms in a food web with an introduced omnivore
Source: Ecol Evol. 2017 Jun 13;7(13):5016–25. doi: 10.1002/ece3.2773 (PMC5496542; doi:10.1002/ece3.2773)
Supplement: Supplementary file 1 [file ECE3-7-5016-s001.docx]

**Appendix S1**

Supporting information to accompany “Stabilizing mechanisms in a food web with an introduced omnivore.”

*Tables and figures*

| **Table S1.** Average contribution of each taxa and life stage to the average Bray-Curtis dissimilarity. | |
| --- | --- |
| Taxa and life stage | Average contribution |
| *Acartia* nauplii | 0.1943 |
| *Tmora* nauplii | 0.0670 |
| *Acartia* adult | 0.0351 |
| *Acartia* copepodite | 0.0178 |
| *Oithona* adult | 0.0050 |
| *Tmora* copepodite | 0.0028 |
| *Oithona* copepodite | 0.0024 |
| *Tmora* adult | 0.0018 |
| *Harpactecoid* copepodite | 0.0011 |
| *Oithona* nauplii | 0.0003 |

| **Table S2.** Tukey HSD results on copepod density data | | | | | | |  |
| --- | --- | --- | --- | --- | --- | --- | --- |
|  | Farm |  |  |  |  |  | |
| Reference | 0.0317 |  |  |  |  |  | |
|  |  |  |  |  |  |  | |
|  | Adult | Copopedite | Nauplii |  |  |  | |
| Adult | - | 0.2267 | <0.001 |  |  |  | |
| Copopedite |  | - | <0.001 |  |  |  | |
| Nauplii |  |  | - |  |  |  | |
|  |  |  |  |  |  |  | |
|  | Reference - adult | Reference - copopedite | Reference - nauplii | Farm - adult | Farm - copopedite | Farm - nauplii | |
| Reference - adult | - | 0.7018 | <0.001 | 0.9606 | 0.4776 | <0.001 | |
| Reference - copopedite |  | - | 2.387E-06 | 9.856E-01 | 9.984E-01 | 7.871E-05 | |
| Reference - nauplii |  |  | - | 4.671E-06 | 1.599E-06 | 8.967E-02 | |
| Farm - adult |  |  |  | - | 0.8976 | <0.001 | |
| Farm - copopedite |  |  |  |  | - | 4.715E-05 | |
| Farm - nauplii |  |  |  |  |  | - | |

| **Table S3.** Stable isotope data for the three tissue types analysed in this study. | | | | | | | | | |
| --- | --- | --- | --- | --- | --- | --- | --- | --- | --- |
| Date collected | Tissue^1^ | Site | Length | Total dry weight | Delta 15N | Percent nitrogen | Delta 13C | Delta 13C corrected | Percent carbon |
| 02-Sep-10 | M | 1+ | 17 | 0.77 | 5.74 | 8.48 | -20.79 | -19.83 | 36.7 |
| 02-Sep-10 | M | 1+ | 19.9 | 0.72 | 5.95 | 8.45 | -20.11 | -19.51 | 33.47 |
| 24-Aug-10 | M | 1+ | 21 | 0.54 | 5.24 | 9.42 | -20.23 | -19.79 | 35.73 |
| 02-Sep-10 | M | 1+ | 21.6 | 0.9 | 5.31 | 8.51 | -20.59 | -19.9 | 34.48 |
| 24-Aug-10 | M | 1+ | 24.1 | 1.07 | 5.36 | 8.68 | -20.06 | -19.4 | 34.83 |
| 24-Aug-10 | M | 1+ | 25.7 | 0.92 | 5.41 | 8.13 | -20.58 | -19.97 | 32.26 |
| 24-Aug-10 | M | 1+ | 29.5 | 0.134 | 5.43 | 7.49 | -22.54 | -20.17 | 43.09 |
| 24-Aug-10 | M | 1+ | 33.7 | 0.572 | 5.64 | 0.52 | -22.13 | -20.67 | 2.53 |
| 24-Aug-10 | M | 1+ | 35.1 | 0.88 | 5.91 | 9.43 | -21.25 | -19.79 | 45.53 |
| 24-Aug-10 | M | 1+ | 36 | 0.881 | 5.67 | 9.3 | -21.58 | -20.2 | 44.1 |
| 02-Sep-10 | M | 1+ | 37.45 | 0.856 | 6.47 | 8.32 | -21.2 | -19.84 | 39.32 |
| 02-Sep-10 | M | 1+ | 37.47 | 0.945 | 6.41 | 9.95 | -20.35 | -19.7 | 39.88 |
| 24-Aug-10 | M | 1+ | 37.6 | 0.96 | 5.92 | 8.18 | -21.63 | -19.7 | 43.34 |
| 02-Sep-10 | M | 1+ | 37.93 | 0.944 | 6.85 | 5.35 | -20.98 | -19.87 | 23.94 |
| 24-Aug-10 | M | 1+ | 39.3 | 1.01 | 5.94 | 8.52 | -21.86 | -19.8 | 46.34 |
| 24-Aug-10 | M | 1+ | 40 | 0.9 | 6.47 | 9 | -20.95 | -19.51 | 43.29 |
| 02-Sep-10 | M | 1+ | 40.02 | 0.933 | 6.51 | 6.29 | -20.83 | -19.53 | 29.32 |
| 24-Aug-10 | M | 1+ | 40.1 | 0.841 | 6.13 | 9.18 | -21.91 | -20.08 | 47.71 |
| 02-Sep-10 | M | 1+ | 40.1 | 1.01 | 5.88 | 7.85 | -21.72 | -19.65 | 42.72 |
| 02-Sep-10 | M | 1+ | 40.33 | 0.922 | 6.5 | 7.34 | -20.81 | -19.56 | 33.87 |
| 24-Aug-10 | M | 1+ | 40.5 | 0.696 | 5.97 | 11.42 | -21.17 | -19.62 | 56.16 |
| 24-Aug-10 | M | 1+ | 40.6 | 0.873 | 6 | 8.08 | -21.41 | -19.38 | 43.61 |
| 24-Aug-10 | M | 1+ | 40.8 | 0.792 | 6.23 | 8.47 | -21.75 | -19.77 | 45.4 |
| 24-Aug-10 | M | 1+ | 40.94 | 0.732 | 6.5 | 10.38 | -20.35 | -19.63 | 42.36 |
| 02-Sep-10 | M | 1+ | 41.11 | 0.894 | 6.61 | 9.47 | -20.64 | -19.77 | 40.01 |
| 24-Aug-10 | M | 1+ | 41.57 | 0.879 | 6.62 | 9.49 | -20.59 | -19.62 | 41.12 |
| 02-Sep-10 | M | 1+ | 41.94 | 0.982 | 6.43 | 8.27 | -20.88 | -19.55 | 38.88 |
| 24-Aug-10 | M | 1+ | 42.6 | 1.018 | 6.27 | 9.35 | -21.52 | -20.08 | 44.91 |
| 02-Sep-10 | M | 1+ | 42.6 | 0.95 | 6.19 | 7.28 | -21.32 | -19.08 | 40.86 |
| 24-Aug-10 | M | 1+ | 42.9 | 0.916 | 6.01 | 7.71 | -22.12 | -19.66 | 45.03 |
| 24-Aug-10 | M | 1+ | 43.6 | 0.61 | 6.59 | 8.26 | -20.27 | -19.68 | 32.57 |
| 02-Sep-10 | M | 1+ | 43.96 | 0.938 | 6.53 | 5.36 | -20.79 | -19.59 | 24.52 |
| 24-Aug-10 | M | 1+ | 44.08 | 0.909 | 6.44 | 7.14 | -20.88 | -19.74 | 32.15 |
| 24-Aug-10 | M | 1+ | 44.11 | 0.984 | 6.6 | 9.46 | -21.07 | -19.97 | 42.26 |
| 02-Sep-10 | M | 1+ | 44.12 | 0.994 | 6.36 | 8.24 | -20.94 | -19.88 | 36.42 |
| 24-Aug-10 | M | 1+ | 44.2 | 0.908 | 5.78 | 9.24 | -21.52 | -20.08 | 44.4 |
| 24-Aug-10 | M | 1+ | 44.3 | 0.998 | 5.98 | 8.25 | -21.04 | -20.1 | 35.52 |
| 24-Aug-10 | M | 1+ | 44.6 | 1.068 | 5.81 | 8.11 | -21.76 | -19.35 | 46.94 |
| 24-Aug-10 | M | 1+ | 44.98 | 0.887 | 6.27 | 8.82 | -20.74 | -19.66 | 39.28 |
| 24-Aug-10 | M | 1+ | 44.99 | 0.917 | 6.45 | 8.81 | -20.73 | -19.74 | 38.38 |
| 24-Aug-10 | M | 1+ | 46.24 | 0.915 | 6.76 | 8.99 | -20.63 | -19.73 | 38.31 |
| 24-Aug-10 | M | 1+ | 46.7 | 0.835 | 6.2 | 7.81 | -21.39 | -19.23 | 43.29 |
| 02-Sep-10 | M | 1+ | 46.79 | 0.816 | 6.34 | 8.1 | -21.22 | -20.1 | 36.33 |
| 24-Aug-10 | M | 1+ | 46.9 | 0.937 | 5.47 | 9.11 | -21.24 | -20.21 | 40.07 |
| 02-Sep-10 | M | 1+ | 47.4 | 1.06 | 6.81 | 9.34 | -20.99 | -20.04 | 40.34 |
| 24-Aug-10 | M | 1+ | 47.55 | 0.95 | 6.49 | 10.06 | -20.69 | -19.57 | 45.19 |
| 24-Aug-10 | M | 1+ | 48.56 | 0.89 | 6.4 | 8.32 | -21.19 | -19.46 | 42.44 |
| 24-Aug-10 | M | 1+ | 49 | 0.929 | 5.93 | 8.17 | -21.55 | -19.35 | 45.62 |
| 24-Aug-10 | M | 1+ | 49.02 | 0.976 | 6.59 | 8.95 | -21.03 | -19.92 | 40.12 |
| 02-Sep-10 | M | 1+ | 49.55 | 0.956 | 6.98 | 8.69 | -20.59 | -19.69 | 37.01 |
| 24-Aug-10 | M | 1+ | 49.6 | 0.94 | 6.03 | 7.91 | -20.71 | -19.61 | 35.34 |
| 24-Aug-10 | M | 1+ | 50 | 1.082 | 5.89 | 9.19 | -21.68 | -20.07 | 45.72 |
| 02-Sep-10 | M | 1+ | 54.34 | 0.933 | 6.4 | 8.59 | -21.22 | -19.79 | 41.17 |
| 24-Aug-10 | M | 1+ | 54.5 | 0.771 | 5.88 | 8.8 | -21.07 | -19.63 | 42.34 |
| 02-Sep-10 | M | 1+ | 54.72 | 0.94 | 6.64 | 8.73 | -20.71 | -19.69 | 38.24 |
| 02-Sep-10 | M | 1+ | 56.78 | 0.904 | 5.99 | 8.73 | -20.31 | -19.57 | 35.74 |
| 02-Sep-10 | M | 1+ | 63.13 | 0.904 | 6.28 | 8.59 | -21.25 | -19.89 | 40.6 |
| 24-Aug-10 | A | 1+ | NA | 0.072 | 7.02 | 10.11 | -20.98 | -19.86 | 45.24 |
| 24-Aug-10 | A | 1+ | NA | 0.066 | 6.33 | 8.91 | -21.24 | -20.08 | 40.3 |
| 24-Aug-10 | A | 1+ | NA | 0.081 | 6.95 | 8.68 | -21.04 | -20.11 | 37.18 |
| 30-Aug-10 | A | 1+ | NA | 0.096 | 7.25 | 3.82 | -21.09 | -19.53 | 17.82 |
| 30-Aug-10 | A | 1+ | NA | 0.099 | 7.4 | 5.94 | -20.47 | -19.85 | 24.14 |
| 30-Aug-10 | A | 1+ | NA | 0.14 | 7.52 | 3.74 | -21.4 | -20.59 | 15.95 |
| 30-Aug-10 | A | 1+ | NA | 0.139 | 8.35 | 5.5 | -21.1 | -20.63 | 22.73 |
| 31-Aug-10 | A | 1+ | NA | 0.162 | 7.68 | 3.38 | -22.2 | -21.98 | 16.37 |
| 31-Aug-10 | A | 1+ | NA | 0.245 | 7.55 | 2.53 | -22.3 | -22.82 | 12.58 |
| 31-Aug-10 | A | 1+ | NA | 0.199 | 7.54 | 2.95 | -22.24 | -21.57 | 13.61 |
| 31-Aug-10 | A | 1+ | NA | 0.163 | 7.19 | 3.06 | -21.79 | -19.42 | 14.57 |
| 31-Aug-10 | A | 1+ | NA | 0.177 | 7.26 | 2.92 | -21.78 | -20.38 | 14.05 |
| 02-Sep-10 | A | 1+ | NA | 0.264 | 8.06 | 2.1 | -22.66 | -24.49 | 10.03 |
| 02-Sep-10 | A | 1+ | NA | 0.256 | 7.45 | 2.92 | -22.44 | -20.67 | 14.21 |
| 02-Sep-10 | A | 1+ | NA | 0.189 | 7.55 | 3.12 | -22.58 | -20.77 | 16.18 |
| 24-Aug-10 | N | 1+ | NA | 0.183 | 6.03 | 8.17 | -24.01 | -20.21 | 58.51 |
| 24-Aug-10 | N | 1+ | NA | 0.676 | 5.55 | 4.47 | -23.3 | -21.48 | 19.64 |
| 24-Aug-10 | N | 1+ | NA | 0.383 | 5.42 | 8.13 | -23.02 | -21.42 | 34.38 |
| 25-Aug-10 | N | 1+ | NA | 2.096 | 6.45 | 2.07 | -23.38 | -19.61 | 10.15 |
| 25-Aug-10 | N | 1+ | NA | 0.967 | 6.08 | 2.83 | -23.87 | -21.8 | 13.28 |
| 25-Aug-10 | N | 1+ | NA | 1.187 | 6.57 | 2.21 | -23.87 | -19.35 | 13.41 |
| 30-Aug-10 | N | 1+ | NA | 0.833 | 5.83 | 3.64 | -22.98 | -21.93 | 14.51 |
| 30-Aug-10 | N | 1+ | NA | 0.62 | 5.46 | 2.31 | -24.12 | -22.15 | 19.67 |
| 30-Aug-10 | N | 1+ | NA | 0.179 | 5.42 | 10.63 | -23.05 | -22.15 | 70.72 |
| 30-Aug-10 | N | 1+ | NA | 0.204 | 4.81 | 8.11 | -23.89 | -20.64 | 76.04 |
| 31-Aug-10 | N | 1+ | NA | 1.95 | 6.33 | 1.04 | -23.28 | -21.53 | 7.68 |
| 31-Aug-10 | N | 1+ | NA | 1.751 | 6.2 | 1.49 | -22.68 | -21.57 | 9.28 |
| 31-Aug-10 | N | 1+ | NA | 2.215 | 6.02 | 0.81 | -23.4 | -19.85 | 7.68 |
| 31-Aug-10 | N | 1+ | NA | 2.043 | 5.93 | 0.66 | -23.59 | -18.85 | 7.56 |
| 31-Aug-10 | N | 1+ | NA | 1.268 | 5.65 | 1.3 | -23.24 | -20.33 | 11.76 |
| 02-Sep-10 | N | 1+ | NA | 0.646 | 5.46 | 2.07 | -23.64 | -20.96 | 19.55 |
| 02-Sep-10 | N | 1+ | NA | 2.026 | 5.69 | 1.23 | -22.75 | -21.77 | 7.59 |
| 02-Sep-10 | N | 1+ | NA | 2.187 | 6.48 | 1.29 | -23.12 | -21.97 | 7.91 |
| 24-Aug-10 | S | 1+ | NA | 0.623 | 4.64 | 2.41 | -23.87 | -22.01 | 16.45 |
| 24-Aug-10 | S | 1+ | NA | 0.889 | 3.63 | 1.8 | -23.96 | -22.27 | 11.78 |
| 24-Aug-10 | S | 1+ | NA | 1.169 | 2.71 | 1.21 | -24.12 | -22.36 | 8.29 |
| 25-Aug-10 | S | 1+ | NA | 1.406 | 3.44 | 1.04 | -24.36 | -22.49 | 7.17 |
| 25-Aug-10 | S | 1+ | NA | 1.304 | 3.44 | 1.11 | -24.43 | -22.44 | 7.83 |
| 30-Aug-10 | S | 1+ | NA | 1.067 | 3.68 | 1.56 | -24.69 | -21.89 | 11.87 |
| 30-Aug-10 | S | 1+ | NA | 1.475 | 3.18 | 1.01 | -24.69 | -21.9 | 7.83 |
| 30-Aug-10 | S | 1+ | NA | 1.095 | 3.12 | 1.43 | -24.98 | -21.82 | 11.58 |
| 30-Aug-10 | S | 1+ | NA | 1.257 | 3.72 | 1.2 | -25.28 | -22.14 | 9.73 |
| 30-Aug-10 | S | 1+ | NA | 1.227 | 3.04 | 1.18 | -24 | -22.13 | 8.13 |
| 31-Aug-10 | S | 1+ | NA | 1 | 3.28 | 1.62 | -25.47 | -22.15 | 13.27 |
| 31-Aug-10 | S | 1+ | NA | 0.824 | 2.77 | 1.84 | -25.23 | -22.72 | 13.75 |
| 31-Aug-10 | S | 1+ | NA | 1.02 | 3.04 | 1.47 | -25.34 | -22.39 | 11.64 |
| 31-Aug-10 | S | 1+ | NA | 1.138 | 3.91 | 1.17 | -25.44 | -22.08 | 9.99 |
| 02-Sep-10 | M | 2+ | 15.5 | 0.57 | 5.66 | 9.97 | -21.29 | -20.92 | 37.15 |
| 02-Sep-10 | M | 2+ | 21.6 | 0.87 | 6.09 | 10.21 | -20.83 | -20.3 | 39.76 |
| 02-Sep-10 | M | 2+ | 24.8 | 0.67 | 6.36 | 9.88 | -20.82 | -20.41 | 37.27 |
| 24-Aug-10 | M | 2+ | 26.6 | 0.99 | 6.07 | 9.75 | -20.85 | -20.1 | 40.14 |
| 24-Aug-10 | M | 2+ | 27 | 0.72 | 5.95 | 9.03 | -20.86 | -20.14 | 36.93 |
| 24-Aug-10 | M | 2+ | 30.1 | 0.61 | 5.86 | 9.37 | -20.97 | -20.37 | 37.05 |
| 02-Sep-10 | M | 2+ | 36.8 | 0.939 | 6.75 | 9.46 | -21.14 | -20.17 | 40.99 |
| 24-Aug-10 | M | 2+ | 37.68 | 0.958 | 6.53 | 10.32 | -21.15 | -20.33 | 43.16 |
| 02-Sep-10 | M | 2+ | 38.02 | 0.888 | 6.49 | 8.98 | -21.21 | -19.88 | 42.13 |
| 24-Aug-10 | M | 2+ | 38.39 | 0.825 | 6.74 | 10.57 | -21.19 | -20.3 | 44.96 |
| 02-Sep-10 | M | 2+ | 38.9 | 0.922 | 6.56 | 8.45 | -21.12 | -19.98 | 38.01 |
| 02-Sep-10 | M | 2+ | 39.24 | 0.896 | 6.49 | 7.49 | -21.39 | -19.7 | 37.84 |
| 02-Sep-10 | M | 2+ | 40.5 | 0.95 | 6.51 | 7.09 | -21.36 | -19.4 | 37.84 |
| 02-Sep-10 | M | 2+ | 40.53 | 0.853 | 6.56 | 7.32 | -21.78 | -19.95 | 38.1 |
| 24-Aug-10 | M | 2+ | 40.77 | 0.889 | 7.04 | 11.07 | -20.8 | -20.12 | 44.73 |
| 24-Aug-10 | M | 2+ | 40.82 | 0.765 | 6.86 | 9.62 | -20.99 | -20.2 | 40 |
| 24-Aug-10 | M | 2+ | 41.18 | 0.991 | 7.15 | 10.56 | -21.01 | -20.18 | 44.25 |
| 24-Aug-10 | M | 2+ | 41.54 | 0.84 | 6.84 | 11.3 | -20.63 | -20.06 | 44.33 |
| 02-Sep-10 | M | 2+ | 41.91 | 0.857 | 6.55 | 8.42 | -21.17 | -19.05 | 46.19 |
| 24-Aug-10 | M | 2+ | 42.7 | 0.875 | 6.89 | 10.53 | -21.03 | -20.24 | 43.65 |
| 24-Aug-10 | M | 2+ | 42.86 | 0.869 | 7.35 | 10.29 | -21.08 | -20.25 | 43.05 |
| 02-Sep-10 | M | 2+ | 43.21 | 0.896 | 6.72 | 7.72 | -21.46 | -19.75 | 39.28 |
| 02-Sep-10 | M | 2+ | 44.67 | 0.983 | 6.48 | 7.84 | -21.4 | -19.78 | 39.19 |
| 24-Aug-10 | M | 2+ | 45.49 | 1.001 | 6.95 | 9.4 | -21.41 | -20.13 | 43.65 |
| 02-Sep-10 | M | 2+ | 45.55 | 0.938 | 6.76 | 8.36 | -20.82 | -20.03 | 34.74 |
| 24-Aug-10 | M | 2+ | 52.5 | 0.74 | 6.56 | 8.94 | -20.9 | -19.72 | 40.64 |
| 24-Aug-10 | M | 2+ | 54.2 | 0.927 | 7 | 9.39 | -21.34 | -19.95 | 44.63 |
| 24-Aug-10 | M | 2+ | 56.5 | 0.732 | 5.62 | 10.05 | -20.89 | -20.01 | 42.65 |
| 24-Aug-10 | M | 2+ | 60 | 0.942 | 6.06 | 8.78 | -21.07 | -19.83 | 40.43 |
| 24-Aug-10 | M | 2+ | 60 | 0.879 | 6.73 | 8.8 | -21.11 | -20.11 | 38.34 |
| 24-Aug-10 | M | 2+ | 60.2 | 0.67 | 7.1 | 9.44 | -21.07 | -20.2 | 40.03 |
| 24-Aug-10 | M | 2+ | 61.4 | 0.956 | 6.2 | 8.82 | -21.58 | -20.24 | 41.45 |
| 24-Aug-10 | M | 2+ | 61.9 | 0.99 | 6.75 | 8.24 | -22.04 | -20.49 | 40.5 |
| 02-Sep-10 | M | 2+ | 62.76 | 0.932 | 7.25 | 7.09 | -21.51 | -19.59 | 37.5 |
| 24-Aug-10 | M | 2+ | 62.8 | 0.907 | 6.76 | 9.2 | -21.62 | -20.13 | 44.69 |
| 24-Aug-10 | M | 2+ | 62.9 | 0.859 | 6.17 | 8.39 | -21.36 | -19.6 | 43.05 |
| 24-Aug-10 | M | 2+ | 65.1 | 0.944 | 6.4 | 7.92 | -21.33 | -19.21 | 43.53 |
| 24-Aug-10 | M | 2+ | 65.5 | 0.823 | 6.38 | 7.55 | -21.23 | -19.83 | 35.98 |
| 24-Aug-10 | M | 2+ | 66 | 0.93 | 6.32 | 7.87 | -21.1 | -19.1 | 42.31 |
| 24-Aug-10 | M | 2+ | 66 | 1.32 | 6.63 | 7.73 | -22.5 | -19.88 | 46.37 |
| 24-Aug-10 | M | 2+ | 67 | 0.803 | 6.65 | 9.11 | -21.23 | -19.78 | 43.9 |
| 02-Sep-10 | M | 2+ | 67 | 1.42 | 7.06 | 10 | -21.74 | -20.23 | 48.82 |
| 02-Sep-10 | M | 2+ | 67.15 | 0.936 | 7.24 | 7.02 | -22.27 | -19.82 | 40.88 |
| 02-Sep-10 | M | 2+ | 67.5 | 0.69 | 8.14 | 7.44 | -23.45 | -19.76 | 52.67 |
| 24-Aug-10 | M | 2+ | 67.7 | 0.954 | 6.65 | 8.19 | -21.26 | -19.93 | 38.38 |
| 24-Aug-10 | M | 2+ | 68.2 | 0.904 | 6.42 | 8.59 | -21.13 | -19.41 | 43.77 |
| 02-Sep-10 | M | 2+ | 68.82 | 0.939 | 6.68 | 7.15 | -21.61 | -19.9 | 36.36 |
| 24-Aug-10 | M | 2+ | 69.6 | 0.798 | 6.7 | 8.52 | -21.56 | -19.97 | 42.29 |
| 24-Aug-10 | M | 2+ | 70.6 | 0.955 | 6.53 | 8.67 | -21.31 | -19.66 | 43.46 |
| 24-Aug-10 | M | 2+ | 71 | 0.774 | 6.92 | 8.67 | -21.81 | -19.92 | 45.67 |
| 24-Aug-10 | M | 2+ | 71.1 | 0.866 | 6.48 | 8.81 | -21.39 | -19.85 | 43.27 |
| 02-Sep-10 | M | 2+ | 73.38 | 1.236 | 6.85 | 5.31 | -21.58 | -19.86 | 27.04 |
| 24-Aug-10 | M | 2+ | 75.4 | 0.871 | 6.36 | 7 | -21.07 | -18.35 | 42.66 |
| 02-Sep-10 | M | 2+ | 75.88 | 0.949 | 7.15 | 6.9 | -21.35 | -19.73 | 34.42 |
| 24-Aug-10 | M | 2+ | 76 | 0.97 | 6.64 | 6.97 | -21.19 | -19.3 | 36.67 |
| 02-Sep-10 | M | 2+ | 76.12 | 0.972 | 6.76 | 14.22 | -21.23 | -19.57 | 71.52 |
| 02-Sep-10 | M | 2+ | 76.17 | 0.877 | 6.1 | 5.32 | -21.85 | -19.89 | 28.35 |
| 02-Sep-10 | M | 2+ | 76.33 | 0.777 | 6.93 | 5.8 | -22.07 | -20.01 | 31.51 |
| 24-Aug-10 | M | 2+ | 76.4 | 0.896 | 6.57 | 8.62 | -21.76 | -19.77 | 46.18 |
| 02-Sep-10 | M | 2+ | 76.91 | 0.916 | 6.64 | 7.79 | -22.01 | -19.8 | 43.53 |
| 02-Sep-10 | M | 2+ | 77.54 | 0.964 | 7.02 | 7.67 | -21.21 | -19.73 | 37.16 |
| 02-Sep-10 | M | 2+ | 78.96 | 0.828 | 6.9 | 7.31 | -22.11 | -19.84 | 41.29 |
| 02-Sep-10 | M | 2+ | 81.43 | 1.842 | 7.21 | 3.95 | -21.12 | -19.79 | 18.54 |
| 02-Sep-10 | M | 2+ | 82.51 | 0.918 | 7.01 | 7.53 | -21.51 | -19.95 | 37.15 |
| 24-Aug-10 | A | 2+ | NA | 0.084 | 7.06 | 6.78 | -21.2 | -20.01 | 30.87 |
| 24-Aug-10 | A | 2+ | NA | 0.113 | 7.4 | 3.79 | -21.97 | -20.29 | 19.13 |
| 25-Aug-10 | A | 2+ | NA | 1.282 | 7.13 | 0.59 | -21.4 | -21.53 | 1.9 |
| 30-Aug-10 | A | 2+ | NA | 0.083 | 7.56 | 9.91 | -20.82 | -17.12 | 38.93 |
| 30-Aug-10 | A | 2+ | NA | 0.143 | 7.79 | 4.58 | -21.3 | -22.92 | 18.65 |
| 30-Aug-10 | A | 2+ | NA | 0.148 | 7.72 | 4.45 | -20.98 | -22.52 | 17.79 |
| 31-Aug-10 | A | 2+ | NA | 1.075 | 7.68 | 0.84 | -20.68 | -21.3 | 2.28 |
| 31-Aug-10 | A | 2+ | NA | 0.159 | 8.09 | 6.69 | -21.3 | -22.04 | 28.23 |
| 31-Aug-10 | A | 2+ | NA | 0.294 | 7.23 | 2.76 | -21.93 | -24.02 | 13.62 |
| 02-Sep-10 | A | 2+ | NA | 0.249 | 7.56 | 1.83 | -22.8 | -20.94 | 9.59 |
| 02-Sep-10 | A | 2+ | NA | 0.191 | 7.48 | 2.24 | -22.02 | -20.46 | 11.02 |
| 24-Aug-10 | N | 2+ | NA | 0.557 | 5.32 | 3.81 | -23.19 | -20.86 | 20.5 |
| 24-Aug-10 | N | 2+ | NA | 1.313 | 6.32 | 2.17 | -23.15 | -18.3 | 13.19 |
| 24-Aug-10 | N | 2+ | NA | 0.885 | 5.96 | 2.91 | -23.81 | -19.76 | 17.04 |
| 25-Aug-10 | N | 2+ | NA | 1.236 | 5.91 | 3.2 | -22.89 | -21.95 | 11.28 |
| 25-Aug-10 | N | 2+ | NA | 1.497 | 6.21 | 3.07 | -22.76 | -21.16 | 11.33 |
| 25-Aug-10 | N | 2+ | NA | 0.519 | 5.66 | 5.37 | -22.81 | -21.92 | 21.74 |
| 30-Aug-10 | N | 2+ | NA | 0.336 | 6.03 | 6.75 | -22.4 | -21.57 | 41.84 |
| 30-Aug-10 | N | 2+ | NA | 0.746 | 6.12 | 2.54 | -23.01 | -22.39 | 16.18 |
| 30-Aug-10 | N | 2+ | NA | 0.264 | 6.09 | 7.79 | -22.52 | -21.5 | 51.32 |
| 30-Aug-10 | N | 2+ | NA | 0.233 | 6.16 | 6.01 | -23.18 | -21.82 | 47.87 |
| 30-Aug-10 | N | 2+ | NA | 0.272 | 6.15 | 7.27 | -22.65 | -21.69 | 48.09 |
| 31-Aug-10 | N | 2+ | NA | 0.255 | 6.42 | 6.09 | -23.14 | -21.68 | 47.14 |
| 31-Aug-10 | N | 2+ | NA | 0.25 | 6.48 | 6.5 | -23.09 | -21.77 | 48.65 |
| 31-Aug-10 | N | 2+ | NA | 0.716 | 6.24 | 3.38 | -22.65 | -21.2 | 22.63 |
| 31-Aug-10 | N | 2+ | NA | 0.269 | 6.26 | 7.8 | -22.67 | -21.78 | 50.02 |
| 31-Aug-10 | N | 2+ | NA | 1.556 | 6.47 | 1.29 | -23.23 | -19.84 | 11.68 |
| 02-Sep-10 | N | 2+ | NA | 1.811 | 6.65 | 0.7 | -24.25 | -21.18 | 7.05 |
| 02-Sep-10 | N | 2+ | NA | 0.315 | 5.66 | 3.18 | -24.28 | -23.27 | 28.33 |
| 02-Sep-10 | N | 2+ | NA | 1.47 | 6.52 | 0.74 | -24.41 | -20.97 | 8.12 |
| 24-Aug-10 | S | 2+ | NA | 1.354 | 4.15 | 1.15 | -24.17 | -21.1 | 9.21 |
| 24-Aug-10 | S | 2+ | NA | 1.355 | 3.22 | 1.12 | -24.15 | -21.57 | 8.43 |
| 24-Aug-10 | S | 2+ | NA | 1.077 | 3.2 | 1.38 | -24.58 | -22 | 10.44 |
| 25-Aug-10 | S | 2+ | NA | 1.64 | 3.69 | 1.02 | -24.43 | -22.06 | 7.36 |
| 25-Aug-10 | S | 2+ | NA | 1.066 | 3.32 | 1.27 | -23.52 | -22.13 | 8.27 |
| 25-Aug-10 | S | 2+ | NA | 1.456 | 3.32 | 0.89 | -25.04 | -22.04 | 7.33 |
| 30-Aug-10 | S | 2+ | NA | 1.828 | 3.21 | 0.79 | -24.99 | -21.89 | 6.44 |
| 30-Aug-10 | S | 2+ | NA | 1.174 | 3 | 1.21 | -24.83 | -22.42 | 9.06 |
| 30-Aug-10 | S | 2+ | NA | 1.313 | 3.23 | 1.11 | -24.75 | -22.26 | 8.31 |
| 30-Aug-10 | S | 2+ | NA | 1.724 | 3.22 | 0.84 | -24.64 | -22.36 | 6.12 |
| 30-Aug-10 | S | 2+ | NA | 1.474 | 3.18 | 0.98 | -24.37 | -22.81 | 6.44 |
| 31-Aug-10 | S | 2+ | NA | 1.478 | 3.98 | 0.86 | -24.38 | -21.83 | 6.69 |
| 31-Aug-10 | S | 2+ | NA | 1.066 | 3.89 | 1.2 | -24.57 | -22.13 | 9.25 |
| 31-Aug-10 | S | 2+ | NA | 0.842 | 4.2 | 1.43 | -24.84 | -22.45 | 11.09 |
| 31-Aug-10 | S | 2+ | NA | 1.122 | 3.55 | 1.09 | -24.86 | -22.42 | 8.48 |
| 1. A, M, N and S denote adult copepods, mussels, copepod nauplii and seston, respectively. | | | | | | | | | |
